# Three-Dimensional (3D) Mapping and Catheter Ablation for Simultaneous Reverse Typical and Atypical Atrial Flutter: A Case Report

**DOI:** 10.7759/cureus.48948

**Published:** 2023-11-17

**Authors:** Hajar El Ouartassi, Badre El Boussaadani, Raid Faraj, Ibtissam Fellat, Mohamed Cherti

**Affiliations:** 1 Cardiology, Ibn Sina University Hospital Center, Rabat, MAR; 2 Cardiology, Centre Hospitalier Universitaire (CHU) Mohammed VI – Tanger, Tanger, MAR; 3 Cardiology, Mohammed V University, Rabat, MAR; 4 Cardiology, Mohamed V University, Rabat, MAR

**Keywords:** case report, 3d electroanatomic mapping, catheter ablation, cavo-tricuspid isthmus, atrial flutter

## Abstract

Atrial flutter, a common cardiac arrhythmia, is characterized by rapid and regular atrial contractions that result in a characteristic sawtooth pattern on the electrocardiogram. It emerges due to the formation of reentrant electrical circuits within the atria, giving rise to structured, sawtooth-patterned atrial waves as observed on electrocardiography.

We present the case of a 52-year-old female with a medical history of ankylosing spondylitis, dyslipidemia, and a previous surgical closure of an atrial septal defect. The patient developed a rare form of atrial flutter, characterized by two distinct mechanisms: a clockwise isthmus-dependent flutter and an atypical scar-related flutter around the atriotomy scar. In order to effectively address this complex condition, a successful ablation procedure was performed to target both mechanisms.

This case report offers valuable insights into the complexities surrounding the diagnosis and treatment of a complex case characterized by the coexistence of multiple mechanisms of atrial flutter within a single patient. While catheter ablation has demonstrated improved success rates for typical and atypical atrial flutters when occurring in isolation, predicting the prognosis of complex cases continues to pose challenges.

## Introduction

Atrial flutter is a common arrhythmia characterized by rapid, regular atrial depolarizations at a characteristic rate (240-320 beats per minute) with the absence of an isoelectric baseline between deflections, caused by a macro-reentrant circuit usually occurring in the right atrium and more rarely in the left one. There have been many classifications of atrial flutter over the years, but the most practical one is based on isthmus versus non-isthmus dependency [1]. Atrial flutter becomes more prevalent as individuals age, with a higher incidence observed in those aged 60 or above. It is the second most common cardiac arrhythmia after atrial fibrillation [1]. While it is frequently associated with atrial fibrillation, there is comparatively less knowledge about the incidence and prevalence of atrial flutter when compared to atrial fibrillation. In cases where the decision is made to maintain sinus rhythm for patients with atrial flutter, radiofrequency catheter ablation is often the preferred choice over pharmacologic therapy. Timely diagnosis and treatment of atrial flutter are crucial to preventing complications such as stroke, heart failure, and tachycardia-induced cardiomyopathy. We present an unusual occurrence of a patient who exhibited two distinct mechanisms of atrial flutter: a clockwise isthmus-dependent flutter and an atypical scar-related flutter around the atriotomy scar. This case underscores the difficulties encountered during both the diagnosis and treatment approach, emphasizing the complex nature of managing such situations. While there is a substantial body of literature on atrial flutter, the simultaneous presence of both atypical and typical flutter in the same patient has been sparsely documented. Our paper was written according to the CAse REport (CARE) guidelines. [2]

## Case presentation

**Figure 1:**
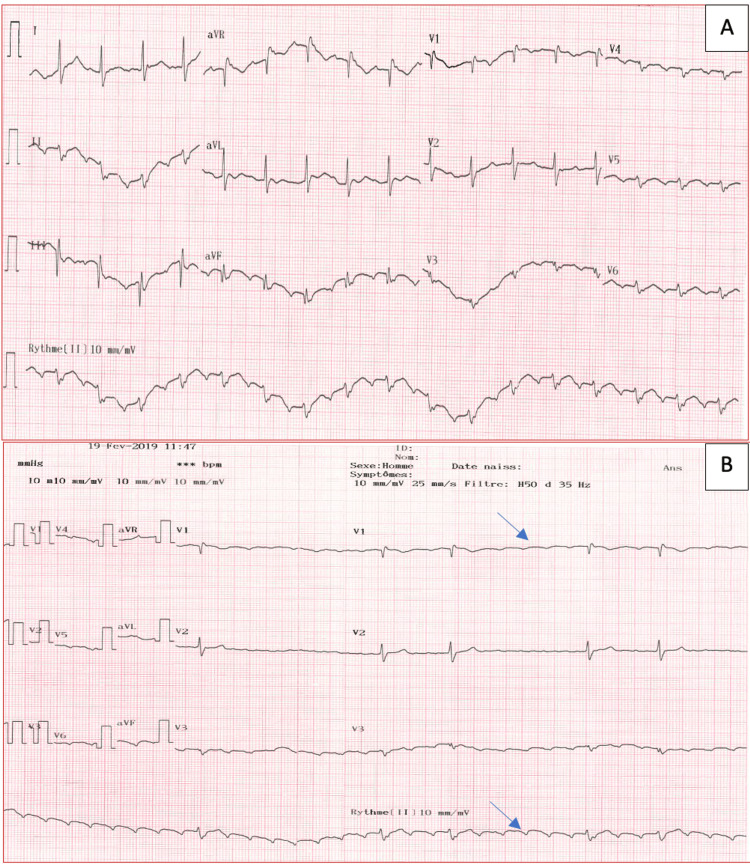
An ECG before (A) and after (B) intravenous administration of adenosine revealing a pattern of atypical atrial flutter with concordant negative flutter waves in the inferior leads and lead V1 (blue arrows).

This is the case of a 52-year-old woman with a medical history of ankylosing spondylitis for 15 years, treated with non-steroidal anti-inflammatory drugs (NSAIDs), dyslipidemia managed with statins, and a surgical history of lateral wall atriotomy for atrial septal defect closure at age 15. The patient presented to the emergency room with palpitations. The initial ECG showed regular tachycardia at 120 beats per minute, which slowed down after intravenous administration of adenosine, revealing an atypical flutter pattern with concordant flutter waves in the inferior leads and lead V1 (Figure 1).

A transthoracic echocardiogram (TTE) was performed and did not show any abnormalities. There was no atrial or ventricular enlargement. The ejection fraction was normal. A cardiac CT scan was performed to assess the coronary arteries and the left atrial anatomy, revealing two pulmonary venous trunks on each side and no left intra-atrial thrombus. There was no evident calcification at the incision site or the site of the repaired atrial septal defect (ASD). The coronary arteries showed minimal calcification with no significant stenosis in the main vessels. Consequently, the patient was admitted for radiofrequency ablation. Under local anesthesia, three guiding sheaths (6-6-8F) were placed in the right femoral vein, followed by the placement of Decapolar exploration catheters (Johnson & Johnson MedTech, New Brunswick, NJ) in the coronary sinus and the lateral right atrial wall. Stimulation induced a mildly sustained flutter, and three-dimensional (3D) mapping with the Decapolar catheter confirmed a typical, clockwise isthmus-dependent flutter (Video 1).

**Video 1:** 3D electroanatomical mapping system with the LAO view showing a typical clockwise isthmus-dependent flutter 3D: three-dimensional; LAO: left anterior oblique

**Figure 2:**
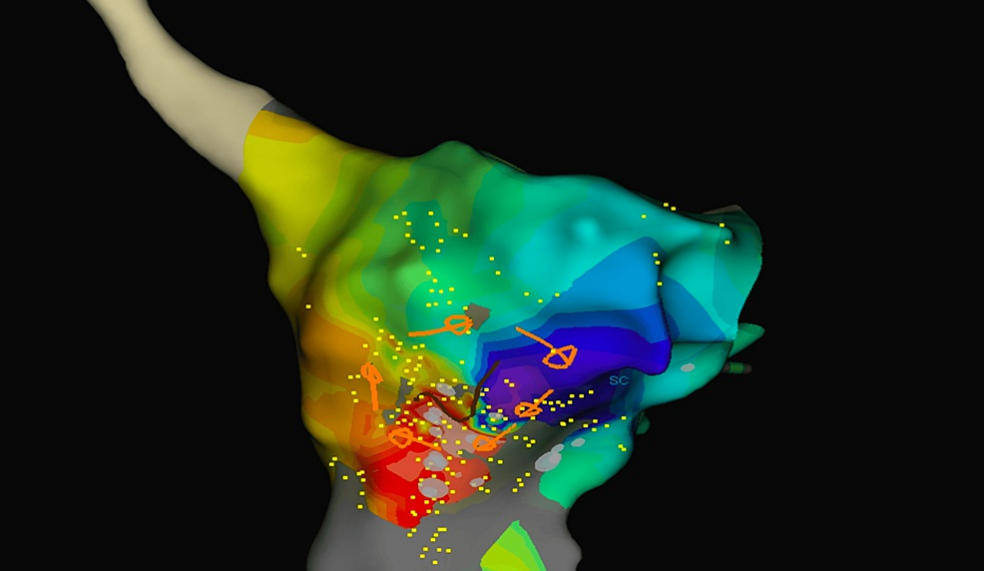
3D electroanatomical mapping system with the RAO view showing a clockwise rotation of atypical atrial flutter with early to late activation around the scar using a color spectrum from red (marking early) to purple (marking late) as an arbitrary reference. 3D: three-dimensional; RAO: right anterior oblique

Radiofrequency ablation was performed at the cavotricuspid isthmus (CTI), resulting in a bidirectional block and termination of the flutter, restoring sinus rhythm. Subsequently, another flutter was induced by stimulation, and 3D mapping revealed a circuit around the atriotomy scar on the lateral wall of the right atrium, involving an isthmus with the tricuspid valve (Figure 2, Video 2).

**Video 2:** 3D electroanatomic activation map of the right atrium showing clockwise rotation of atypical atrial flutter around the atriotomy. 3D: three-dimensional

Ablation from the incisional scar to the tricuspid annulus successfully terminated the flutter, restoring sinus rhythm. No sustained flutter could be induced after the procedure, both at baseline and under the isoproterenol challenge. The procedure was uncomplicated, and a post-ablation ECG showed sinus rhythm with a normal heart rate. In conclusion, a successful ablation was performed for an atypical scar-related flutter around the atriotomy scar resulting from the previous surgical closure of the atrial septal defect. Additionally, a typical isthmus-dependent flutter was also ablated. The patient was discharged on the same day, and during the one-year follow-up, no recurrence of arrhythmia was observed based on ambulatory ECG recordings.

## Discussion

The classification of atrial macro-reentrant arrhythmias is challenging due to inconsistent terminology in the literature and clinical practice. One approach to classifying atrial flutter is to divide it into two main categories based on whether the flutter circuit involves the CTI in the right atrium [3]. Typical atrial flutter is characterized by a macro-reentrant circuit passing through the CTI, a structure located between the inferior vena cava and the tricuspid valve annulus. There are two directions of the circuit: counterclockwise typical (or "common") atrial flutter: This is the most common form, where the circuit proceeds up the atrial septum, down the lateral atrial wall, and through the CTI. It is characterized by continuous, regular negative flutter waves in the inferior leads (II, III, and augmented vector foot (aVF)), often described as a "sawtooth" pattern. Leads I and augmented vector left (aVL) show flat waves, and the atrial deflections in V1 can be positive, biphasic, or negative. In the less common form of clockwise (or reverse) typical atrial flutter, the circuit goes in the opposite direction. The ECG typically shows positive flutter waves in the inferior leads (II, III, and aVF), and a distinct bimodal negative wave in the shape of a W is seen in lead V1. The ECG discordance in the flutter wave direction between the inferior leads and lead V1 is a characteristic feature of typical atrial flutter [4]. On the other hand, atypical atrial flutter can occur in either the right or left atrium and is often associated with structural heart disease, previous surgical procedures, or catheter ablation for atrial fibrillation. It is defined by a reentrant circuit that does not rely on the CTI. However, three types of isthmus-dependent atrial flutter (lower loop, double wave, and intra-isthmus reentry) are also considered atypical atrial flutter [5]. Scar-related right atrial flutter, a form of atypical atrial flutter, usually develops after atriotomy or surgery for congenital heart disease. It often occurs years after the procedure, indicating the need for atrial remodeling for stable reentry around the surgical obstacles [6]. This was the case of our patient, who benefited from the surgical closure of an atrial septal defect at the age of 15. Many years later, their atriotomy scar served as a circuit for atypical atrial flutter. The ECG pattern of this form is highly variable, but flutter waves in the inferior leads and lead V1 are often concordant [4], similar to our case. It is not unusual for two or more ECG patterns to alternate, as CTI-dependent typical flutter not only often coexists with the scar-related atrial flutter, but it is also the most common underlying reentrant circuit in atriotomy patients, which is why the ECG aspect often resembles that of typical atrial flutter [5,7].

Radiofrequency catheter ablation is the preferred treatment for atrial flutter, with high success rates for typical flutter [4]. Nevertheless, if this treatment is now a standard approach for typical flutter [8], there are currently no established guidelines for catheter ablation of atypical flutter [6]. Certainly, the use of entrainment mapping techniques in atypical flutter is crucial for identifying reentrant circuits and determining the critical isthmus(es). The additional use of 3D activation mapping is also an excellent modality. In scar-related right atrial flutter, various ablation methods have been suggested depending on the mechanisms of macro-reentrant circuits. For instance, if a reentry circuit revolves around the atriotomy scar, the recommended approach involves establishing linear lesions between the scar and an anatomical barrier such as the vena cava or the tricuspid annulus. Conversely, if the reentry circuit is located within the scar itself, a focal ablation technique targeting specific conducting channels is recommended [5]. Procedural success can be described as achieving three outcomes: firstly, the cessation of atypical atrial flutter; secondly, establishing a reliable and persistent conduction block across the critical tachycardia isthmus; and thirdly, ensuring that atypical atrial flutter is not induced [5]. In our patient, the presence of lesion-related atypical flutter was revealed by the change in the activation sequence and ECG after ablation of typical atrial flutter. In complex cases like ours, both circuits (typical and atypical) need to be ablated for clinical control of recurrent atrial arrhythmias [9]. Moreover, CTI ablation may facilitate the mapping and ablation of atypical macro-reentry circuits. Therefore, it should be considered in cases of atypical right atrial flutter, even if typical flutter is not present, as this will help prevent its later appearance. While the success rates of radiofrequency ablation of scar-related right atrial flutters have improved over the years, the prognosis of complex cases remains difficult to predict [5, 9].

## Conclusions

This case underscores the challenges of diagnosing and treating complex arrhythmias, especially when multiple mechanisms coexist within the same patient. While catheter ablation has shown promising success rates for typical and atypical atrial flutters when they occur in isolation, the prognosis of complex cases like this one remains unpredictable. While catheter ablation holds promise as an effective treatment, further research and clinical experience are needed to better understand and improve the outcomes of complex atrial flutter cases.
